# Bystander signaling in *C. elegans:* proton microbeam studies

**DOI:** 10.1093/jrr/rrt178

**Published:** 2014-03

**Authors:** Gregory Nelson, Tamako Jones, Leticia Ortloff, John Ford, Delia Nuñez, Leslie Braby

**Affiliations:** 1 Division of Radiation Research, Department of Basic Sciences, Loma Linda University, 11175 Campus St., CSP-A1010, Loma Linda, CA 92354, USA; 2 Department of Nuclear Engineering, Texas A&M University, College Station, TX 77843, USA

## Abstract

**Biological model:** In this project, we investigated the control of radiation-induced genotoxic damage expression in somatic cells of the nematode *Caenorhabditis elegans*. We measured genotoxic damage in the *C. elegans* intestine by irradiating young larvae with 20 intestinal cells. Fourteen of these cells undergo exactly one nuclear division without cytoplasmic division leading to 14 binucleate cells. This nuclear division is synchronized and occurs at the first larval molt. Irradiation induces chromosome aberrations including dicentrics which we can quantify as stable anaphase bridges in the binucleate cells of young adult intestines. The endpoint is dose- and LET-dependent and we have demonstrated that individual intestinal cells have unique radiosensitivities.

**Results:** The project has two components, a genetic screen for genes that control cell sensitivity and a microbeam component to directly probe individual cells. The genetic screen has identified several genes in NHEJ repair and telomere metabolism that modulate overall bridge frequency. Knockout mutants of *cku-70*, *cku-80* and *lig-*4 greatly sensitize animals for anaphase bridge induction. A statistical method was used to determine whether induction of bridges was strictly random and cell autonomous and we determined that expression of bridges in pairs of cells was, in fact, non-random which suggested that signaling between cells affected the pattern of bridge expression. This allowed us to conduct an RNAi and mutation screen for genes that control the signaling (block non-random distributions) and several candidates have been identified.

To directly test the notion that signaling of genotoxic damage occurs, we conducted experiments with alpha particles collimated through slits in metal foils and showed that genotoxic damage could be expressed many cell diameters away from a partial body exposure site. Thus, an *in vivo* bystander effect was demonstrated. Dose targeting was then improved to small regional exposures and eventually to individual cell targeting using 2 MeV protons from the microbeam facility at Texas A&M University. We now employ a green fluorescent protein (GFP)-expressing transgenic worm (*rrIs1[elt-2::GFP]*) to target GFP-positive gut cells via the gut-specific transcription factor *elt-2*. This allows alignment of the cell of interest over the microbeam aperture under appropriate fluorescence illumination.

Microbeam irradiation experiments for many pairwise combinations of cell signal *transmission* and *reception* (observed as expression of anaphase bridges) have been conducted and several interesting patterns emerge. (i) The signaling pattern is cell-specific and does not simply reflect cell–cell distance or require direct contact between cell pairs. (ii) The signal range can be as far as from cell pair 2 to cell pair 8 (>100 µm). (iii) There appears to be a functional compartment boundary at the pharynx/intestine valve as even high-dose exposures to the posterior pharyngeal bulb fail to induce bridges in nearby intestinal cells. (iv) The frequency of signal transmission and reception corresponds broadly to the overall frequency of bridges observed during whole-body irradiations which suggests that direct irradiation and ‘out-of-field’ effects may be additive. These patterns have been analyzed in terms of a cellular logic circuit map for signal transmission and reception.

A dose–response for a subset of microbeam-targeted cells was measured over the range of 5–20 Gy. Controlled cell pair targeting was used to test the potential additivity of signals and we found that effects were supra-additive. Finally, preliminary measurements were conducted on GFP-expressing transgenic strains that bore *cku-70(tm1524)* III and *smk-1(mn156)* V mutations which confer enhanced radiosensitivity. *Cku-70* is a Ku-70 ortholog while *smk-1* is orthologous to the mammalian and *Dictyostelium discoideum* SMEK (suppressor of MEK null) protein. In the *cku-70(0/0)* strain, the severity of the bridges in bystander cells was enhanced, suggesting that signal recipient cells employ NHEJ repair pathways in the expression of anaphase bridges.

Clinical trial registration number: Not applicable.

